# The Formation of Sex Chromosomes in *Silene latifolia* and *S. dioica* Was Accompanied by Multiple Chromosomal Rearrangements

**DOI:** 10.3389/fpls.2020.00205

**Published:** 2020-02-28

**Authors:** Václav Bačovský, Radim Čegan, Denisa Šimoníková, Eva Hřibová, Roman Hobza

**Affiliations:** ^1^Department of Plant Developmental Genetics, Institute of Biophysics of the Czech Academy of Sciences, Brno, Czechia; ^2^Institute of Experimental Botany, Czech Academy of Sciences, Centre of the Region Haná for Biotechnological and Agricultural Research, Olomouc, Czechia

**Keywords:** chromosome painting, double-translocation, pseudo-autosomal region, *Silene*, Y chromosome

## Abstract

The genus *Silene* includes a plethora of dioecious and gynodioecious species. Two species, *Silene latifolia* (white campion) and *Silene dioica* (red campion), are dioecious plants, having heteromorphic sex chromosomes with an XX/XY sex determination system. The X and Y chromosomes differ mainly in size, DNA content and posttranslational histone modifications. Although it is generally assumed that the sex chromosomes evolved from a single pair of autosomes, it is difficult to distinguish the ancestral pair of chromosomes in related gynodioecious and hermaphroditic plants. We designed an oligo painting probe enriched for X-linked scaffolds from currently available genomic data and used this probe on metaphase chromosomes of *S. latifolia* (2n = 24, XY), *S. dioica* (2n = 24, XY), and two gynodioecious species, *S. vulgaris* (2n = 24) and S. *maritima* (2n = 24). The X chromosome-specific oligo probe produces a signal specifically on the X and Y chromosomes in *S. latifolia* and *S. dioica*, mainly in the subtelomeric regions. Surprisingly, in *S. vulgaris* and *S. maritima*, the probe hybridized to three pairs of autosomes labeling their p-arms. This distribution suggests that sex chromosome evolution was accompanied by extensive chromosomal rearrangements in studied dioecious plants.

## Introduction

The genus *Silene* is a model system for sex chromosome evolution, including about 700 species varying greatly in their mating system, ecology and sex determination (Bernasconi et al., 2009). Inside the genus two groups are considered as invaluable for the study of sex chromosome evolution and sex determination; section *Melandrium* and subsection *Otites* (reviewed in Vyskot and Hobza, 2015). Two dioecious plants *S. latifolia* (24, XY) and *S. dioica* (24, XY) from *Melandrium* have heteromorphic sex chromosomes and sex determination similar to mammals (Ming et al., 2007; Charlesworth, 2016). In contrast, related gynodioecious species *S. vulgaris* and *S. maritima* with the same number of autosomes (2n = 24), possess no sex chromosomes having a smaller genome compared to *S. latifolia* or *S. dioica* (Runyeon and Prentice, 1997; Charlesworth and Laporte, 1998; Široký et al., 2001; Stone et al., 2017).

It is generally accepted that the sex chromosomes are derived from an ordinary pair of autosomes (reviewed in Bachtrog, 2006). As a result of suppressed recombination and accumulation of deleterious mutations, the sex chromosomes differ in their structure, function and gene density. The X chromosome becomes hemizygous and X hemizygosity in males leads to special regulatory mechanisms to equalize the transcription ratio between the X chromosomes and autosomes (Charlesworth and Charlesworth, 2005; Muyle et al., 2017; Darolti et al., 2019). As a result of accumulation of deleterious mutations, the Y chromosome is degenerated and the sex chromosomes may differ even within closely related species as demonstrated in human and chimpanzee (Hughes et al., 2010). Interestingly, newly formed sex chromosomes show the same signs of sex chromosome evolutionary pathways, as described in *Drosophila* (Bachtrog et al., 2009) or stickleback species (Yoshida et al., 2014) in which the ancestral Y chromosome fused with an autosome.

In *S. latifolia* and *S. dioica*, sequence data showed that the sex chromosomes evolved from one pair of autosomes with the divergence of X and Y homologous sequences <10 million years ago (Filatov, 2005), estimating the age of older and younger strata (non-recombining part of the sex chromosomes that differ from each other in level of divergence) around 11 and 6.32 mya (Krasovec et al., 2018). The sex chromosomes in *S. latifolia* and *S. dioica*, vary greatly in size having Y chromosome 1.4 larger than X (heteromorphism) (Vyskot and Hobza, 2015). The Y chromosome has a large non-recombining region and the size of the PAR (pseudo-autosomal region) is less than 10% of its chromosome length (Filatov et al., 2009). Both sex chromosomes accumulated various transposable elements (Bergero et al., 2008b; Kubat et al., 2014) and satellites (Cermak et al., 2008; Kejnovský et al., 2013), and differ in histone modifications and DNA methylation (Rodríguez Lorenzo et al., 2018; Bačovský et al., 2019).

Previous studies suggested that the sex chromosomes in *S. latifolia*, especially the Y chromosome, were derived through multiple rearrangements (Bergero et al., 2008a). Deletion mapping revealed that at least one larger inversion occurred after recombination suppression on the Y chromosome (Zluvova et al., 2005), supported also by findings of four genetically mapped genes between *S. latifolia* and *S. dioica* (Filatov, 2005). Later, Hobza et al. (2007) used physical mapping and confirmed two large inversions on the Y chromosome. These findings were further verified by Y deletion mapping showing that at least one inversion had to have occurred during the formation of the Y chromosome (Kazama et al., 2016), accelerating the recombination suppression (Bergero and Charlesworth, 2009). In contrast, comparative mapping between *S. latifolia* and *S. vulgaris* revealed the existence of one large (SvLG12) and two relatively small (SvLG9, Sv small LG) linkage groups that accompanied the sex chromosomes formation in *S. latifolia* (Bergero et al., 2013; Campos et al., 2017). Yet, it is still not clear what pair(s) of autosomes were included in such translocation and if such linkage groups also exist in other gynodioecious species. Thus, this raises two important questions: if *S. vulgaris* possesses three putative parts of three linkage groups corresponding to the X chromosome in *S. latifolia*, what is the origin of these linkage groups and how many chromosomes were involved in sex chromosome formation?

Recent advances in fluorescence *in situ* hybridization (FISH) experiments have provided a variety of techniques which can be used to study the structure, dynamics and origin of certain loci, chromosomal arms and/or specific chromosomes (reviewed in Cui et al., 2016; Bačovský et al., 2018; Huber et al., 2018). Previous cytogenetic studies in *Silene* species were based mainly on physical mapping of satellite rDNA (Široký et al., 2001), repeats and transposable elements (Cermak et al., 2008; Kejnovsky et al., 2009). Although Lengerova et al. (2004) managed to produce discrete signals using various DNA repeats (satellites, rDNA) and specific BAC clones, this approach proved to be cost ineffective due to the large screening of BACs containing only a low amount of repetitive DNA. As an another option, Hobza et al. (2004) designed a protocol using microdissected X and Y chromosomes from *S. latifolia* for whole chromosome painting. These probes produced relatively discrete signals on both sex chromosomes, but high amount of suppressive unlabeled DNA with very strict hybridization conditions made the use of such method very problematic in other *Silene* species (Hobza and Vyskot, 2007). The recent development of oligo painting probes in plants has proved to be useful in the detection of chromosomal aberrations and in comparative cytogenetics (reviewed in Jiang, 2019). In principal, oligo painting probe can be used to label particular regions containing enough short unique oligo sequences to be computationally isolated, synthesized, pooled and labeled (Han et al., 2015). Such probes, designed from conserved sequences of one species were used e.g. for developing karyotype among genetically related *Solanum* species (Braz et al., 2018), for differentiating of A, B, and D genomes in wheat (Tang et al., 2018), in comparative physical mapping of sex chromosomes in *Populus* (Xin et al., 2018) and in examination of meiotic pairing in polyploid *Solanum* species (He et al., 2018).

In this work, we designed an X chromosome-specific oligo probe enriched by X-linked scaffolds based on the *S. latifolia* female genome. We show that such technique is useful for the detection of discrete signals in sex chromosomes in *S. latifolia* and closely related *S. dioica*. In addition, the probe works well in the related gynodioecious species of *S. vulgaris* and *S. maritima*. Based on our results, we discuss the origin of sex chromosomes from one autosomal pair and the possible application of oligo painting probe in further studies. Our findings support the general hypothesis that multiple chromosomal changes took place during the formation of X and Y chromosomes.

## Materials and Methods

### Plant Material

Seedlings of the *Silene* species listed in Supplementary Table S1 (seeds owned by The Institute of Biophysics of the Czech Academy of Sciences) were used for chromosome preparation following (Bačovský et al., 2019). Young seedlings (average size = 1 cm) were synchronized for 16 h in 1.125 mM hydroxyurea at RT, washed 2× for 5 min in distilled water and incubated 4 h in distilled water at RT. Cells in metaphase were accumulated by 0.05 mM colchicine at RT 4 h. After 4 h, root tips were stored for 16 h in ice cold water according to Pan et al. (1993). This reduced the number of ball metaphases and increased the mitotic index. As a final step, synchronized seedlings were fixed in freshly prepared Clarke’s fixative (ethanol:glacial acetic acid, 3:1, v:v) for 24 h and stored at −20°C in 96% ethanol until use.

### Oligo Painting Probe Selection and Preparation

The oligo painting probe of *S. latifolia*, prepared for X chromosome, was designed using Chorus software as previously described by Han et al. (2015). Briefly, oligo sequences (45 nt; >75% similarity) specific to X chromosome, based on the *S. latifolia* female genome (PRJNA289891; Papadopulos et al., 2015), were selected throughout the X chromosome scaffolds anchored using an X genetic map. Repetitive sequences were discriminated and removed during oligo painting probe design by Chorus pipeline (Han et al., 2015). A total of 12 988 oligo sequences were selected to cover X-linked scaffolds. The oligo sequences were synthesized *de novo* as myTags 20K Immortal library by Arbor Biosciences (Ann Arbor, MI, United States; TATAA Biocenter, Göteborg, Sweden). Labeling and detection of the oligo painting probe followed the published protocol of Han et al. (2015). For labeling of oligo-RNA products, we used universal primers (Eurofins Genomics, Ebersberg, Germany) conjugated with the Cy3 (5′–Cy3–CGTGGTCGCGTCTCA–3′) or primers conjugated with the digoxigenin (5′–DIG CGTGGTCGCGTCTCA–3′), similarly as (Šimoníková et al., 2019). Digoxigenin was detected by FITC conjugated anti-DIG antibody (Roche Life Sciences).

The number of oligo sequences per scaffold, scaffold length, position on genetic map and scaffold ID are included in Supplementary Table S2.

### Chromosome and Probe Preparation

Chromosome spreads were obtained from multiple individuals from one population of studied species listed in Supplementary Table S1. Chromosomes were prepared as described in Bačovský et al. (2019) with minor modifications. Briefly, fixed root tips were washed 2× in distilled water 5 min, 2× in 0.001M citrate buffer 5 min and digested for 45–50 min in 1% enzyme mix (Supplementary Table S3) diluted in 0.001M citrate buffer. Chromosomes were squashed on to slides, freezed in liquid nitrogen and incubated for 5 min in freshly prepared Clarke’s fixative. Prepared slides were used directly for fluorescence *in situ* hybridisation (FISH) or stored at −20°C in 96% ethanol until use.

FISH was performed as described by Schubert et al. (2016) using four different stringencies (Supplementary Table S3). The centromeric *Silene* tandem arrayed repeat (STAR-C) and subtelomeric tandem repeat (X43.1) were used as reference probes described in Bačovský et al. (2019). STAR-C is primarily located in centromeres on the X chromosome and autosomes, and on the Y in an additional two clusters based on stringency conditions (Hobza et al., 2007). Chromosome pictures were captured with Olympus AX70 microscope equipped with the cold cube camera. After image capture, all channels were processed with the software Adobe Photoshop free version CS2. A color histogram for each X and Y chromosome image was drawn using RGB profiler in ImageJ 1.52i FiJi^1^. These histograms display the distribution of DNA probes along each chromosome arm. RGB profiler was used on the same plot, for each type of green, red or blue selection as described in Mathur et al. (2012). The oligo painting probe was used in at least three individual experiments and each labeling pattern was scored in 30 metaphases/interphases per experiment. We did not observe any variation in signal patterns in studied populations.

## Results

### Test for X Chromosome-Specific Oligo Probe Stringency and Oligo Painting Probe Signal Strength

We developed the X chromosome-specific oligo probe from female genomic sequences described in Papadopulos et al. (2015), using the approach described in Han et al. (2015). A total of 12 988 oligo sequences was selected from the entire currently available genomic data (Supplementary Figure S1; Papadopulos et al., 2015), covering on average 2.5–3 oligo sequence/kb (1.8–5.5 oligo sequence/kb) for the selected loci. The total density of selected oligo sequences from the X chromosome is below the recommended level of oligo sequence number per kilobase (0.03 oligo sequence/kb) (Han et al., 2015; Jiang, 2019). Nevertheless, for selected regions an average density of 1.8–5.5 oligo sequence/kb and the average number of oligo sequences is higher than the recommended standard of an oligo painting probe for metaphase chromosomes and single loci, 0.1–0.5 oligo sequence/kb (Han et al., 2015; Jiang, 2019).

**FIGURE 1:**
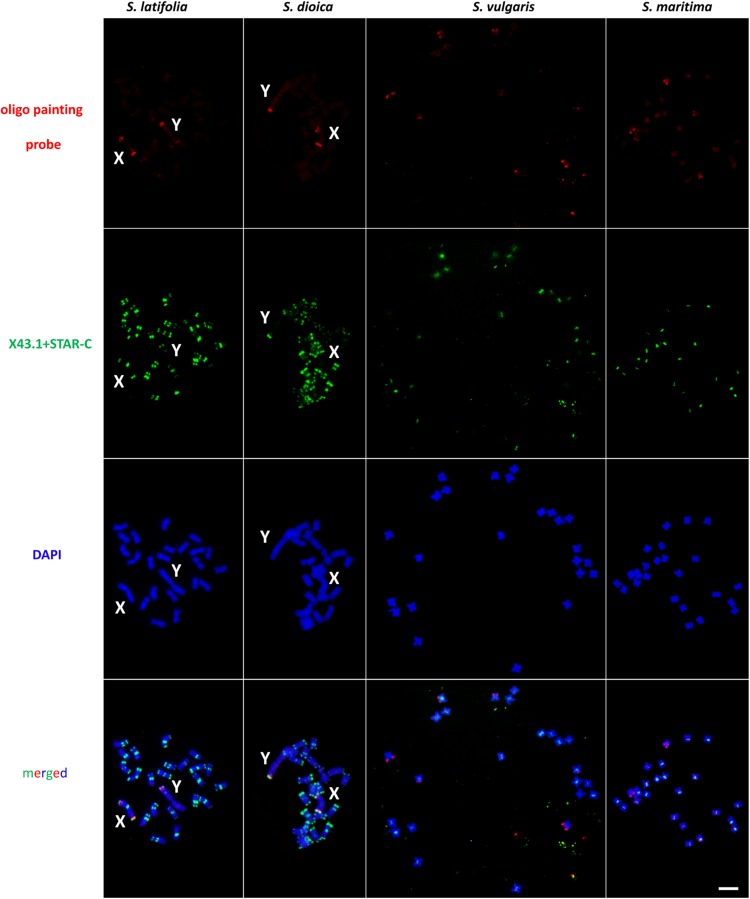
Distribution of X chromosome-specific oligo probe on chromosomes in *Silene latifolia*, *S. dioica*, *S. vulgaris* and *S. maritima*. In *S. latifolia* and *S. dioica*, oligo painting probe has discrete signal only on X and Y chromosomes. In *S. vulgaris* and *S. maritima*, three pairs of autosomes can be differentiated from the whole genome. Scale bar = 5 μm.

To study potential rearrangements accompanying sex chromosome evolution, we used an X chromosome-specific probe in four species in the genus *Silene*, two dioecious (*S. latifolia* and *S. dioica*) and two gynodioecious (*S. vulgaris* and *S. maritima*). In *S. latifolia*, *S. dioica* and *S. vulgaris*, the oligo painting probe yields identical pattern in each species using direct (Cy3-tagged oligo sequences) and indirect labeling (digoxigenin tagged oligo sequences), and different hybridization stringencies (Supplementary Table S3). Only minor changes were observed in signal strength if the amount of oligo painting probe in *S. vulgaris* was increased (set to 1 μg per slide due to the weak overall chromosomal coverage). Nevertheless, 87 and 77% stringency produced very faint signal on the chromosomes of *S. maritima* (data not showed), using direct or indirect labeling and using the same amount of DNA (1 μg of oligo painting probe per slide). Therefore, we tested additional two hybridization stringencies, 68 and 62%, respectively, and we detected a similar pattern in *S. maritima* as in *S. vulgaris* (Figure 1 and Supplementary Figure S3) (signal on three pairs of autosomes). Therefore, 68% hybridization stringency was applied in additional experiments for final analysis in all studied species using 1 μg of X chromosome-specific oligo painting probe per slide (Figure 1 and Supplementary Figure S3).

### X Chromosome-Specific Oligo Probe Pattern in *Silene* Species

**FIGURE 2:**
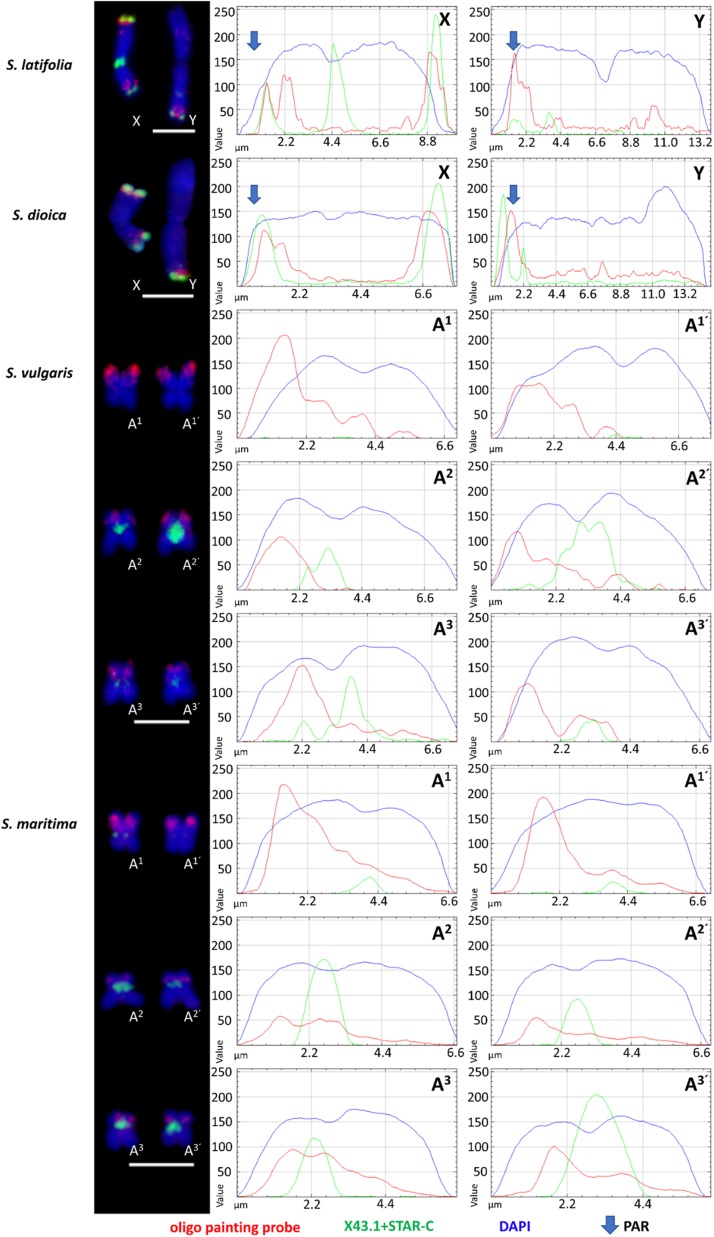
Schematic distribution of X chromosome-specific oligo probe on sex chromosomes in *S. latifolia* and *S. dioica* and individual chromosomes in related gynodioecious species, *S. vulgaris* and *S. maritima*. Note the differences between **X** and **Y** chromosomes in *S. latifolia* and *S. dioica*. In S. *vulgaris* and *S. maritima*, the oligo painting probe signal is located on three pair of autosomes, numbered in this study as **A^1^–A^3^** and **A^1’^–A^3’^**. X43.1, a subtelomeric probe, is presented only on sex chromosomes and autosomes in *S. dioica* and *S. latifolia*. Scale bar = 5 μm.

The designed oligo painting probe hybridized to both ends of the X chromosome arms (including PAR on the p-arm) and to PAR located on Y q-arm in *S. latifolia* and *S. dioica*. In addition, an extra oligo painting probe signal is clearly visible on the X p-arm, suggesting potential gene-rich locus in this (sub)telomeric region (Figure 2). On the Y, the probe colocalizes with X43.1 (sub)telomeric probe band on the Y q-arm (PAR region). The additional oligo painting probe signal was visible on the Y, as an interstitial region, located on the p-arm in both species (Figure 2). We did observe extra (weak) signal on the autosomes, using both Cy3- and digoxigenin-conjugated primers and various hybridization stringencies (77, 68, and 62%). Nevertheless, the extra (weak) signal was affected by low hybridization stringency. In *S. latifolia* and *S. dioica* female karyotype, the oligo painting probe produced the same signal on both X chromosomes as on the X chromosome in males (Supplementary Figure S4). Thus, the X chromosome-specific oligo painting probe used in this work provides a highly reproducible signal in all studied species.

In *S. vulgaris* and *S. maritima*, application of oligo painting probe differentiated three pairs of autosomes, marked in this study as A^1^–A^3’^ (Figure 2). Compared to *S. maritima*, the decrease in hybridization stringency in *S. vulgaris* did not change the number of loci and signals on the chromosomes. In both gynodioecious species, the oligo painting probe labeled almost the entirety of the p-arms of A^1^–A^1’^, including (sub)centromere regions. Additionally, the oligo painting probe had a twofold stronger signal on the first pair of autosomes (A^1^–A^1’^), in *S. vulgaris* and *S. maritima*, than on the second and third (A^2^–A^3’^) autosomal pairs. The oligo painting probe hybridized to subtelomeric (A^2^–A^2’^) or more interstitial regions (A^3^–A^3’^) on these chromosomes (Figures 1, 2).

In interphase, the X chromosome-specific probe differentiated the sex chromosome domains in *S. latifolia* and *S. dioica*, and the A^1^–A^3’^ autosomal regions in *S. vulgaris* and *S. maritima*. In the first two species, the oligo painting probe differentiated two sub-domains located within one nucleus (Supplementary Figures S2a,b). In *S. vulgaris* and *S. maritima*, the oligo painting probe labeled three to six subdomains (Supplementary Figures S2c,d). Despite the average density being 1.8–5.5/kb in selected regions, the total coverage of the whole chromosome is only 0.03 oligo sequence/kb. The lower coverage is apparent (weaker signal) in more relaxed chromatin state in early metaphase/prophase in all studied species (Supplementary Figure S3), showing that the oligo painting probe labeled (sub)telomere of the sex chromosomes and almost half of their length in the autosomes of *S. vulgaris* and *S. maritima*.

## Discussion

**FIGURE 3:**
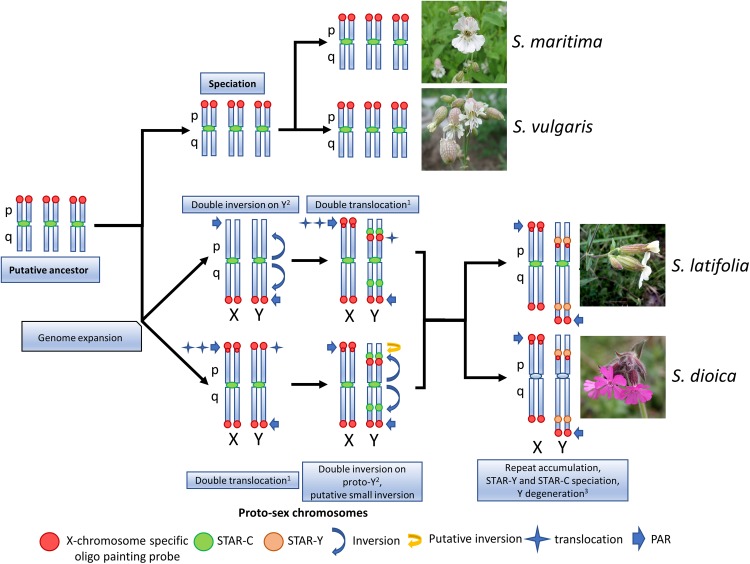
Schematic view of the sex chromosomes evolution in *S. latifolia* and *S. dioica*. The sex chromosomes in *S. latifolia* and *S. dioica* underwent at least one double translocation^1^ (Bergero et al., 2013; Campos et al., 2017) and double inversion^2^ events (Hobza et al., 2007; Zluvova et al., 2007; Bergero et al., 2008a; Kazama et al., 2016). Compared to gynodioecious species (*S. vulgaris* and *S. maritima*), *S. latifolia* and *S. dioica* possess two sex chromosomes having signal from hybridized oligo painting probe. Based on the oligo painting probe signal in this study, two scenarios (supported by the literature) could led to the formation of sex chromosomes in *S. latifolia* and *S. dioica.* If the double translocation moving the segments of STAR-C satellite occurred first, then the autosomal parts have been translocated to proto-sex chromosome X PAR and interstitial Y p-arm region (upper scenario). In the second scenario, the autosomal parts were translocated to the sex chromosomes first, followed by double translocation and at least one putative translocation on the Y p-arm. Flower pictures downloaded on: https://commons.wikimedia.org/wiki/Main_Page.

The oligo painting probe specifically hybridized to pseudo-autosomal region (PAR) located on the Y q-arm and interstitial Y p-arm region, and to both sub-telomeric regions on the X chromosome in *S. latifolia* and *S. dioica*, enriched on the X p-arm sub-telomere. This distribution of the oligo painting probe signal correlates with the pattern of specific histone modifications for active chromatin, namely H3K4me1, H3K4me2, H3K4me3, H3K9ac and gene repressive mark H3K27me3 (Bačovský et al., 2019). Since the Y chromosome still contains many active genes (Bergero and Charlesworth, 2011), the additional band on the X p-arm together with the interstitial Y p-arm region probably represent unique/important gene clusters (Hobza et al., 2018). In related *S. vulgaris* and *S. maritima*, the oligo painting probe clearly differentiates three pairs of autosomes. In these gynodioecious species, the oligo painting probe labels three autosomal p-arms as shown on early metaphase/prophase chromosomes (Supplementary Figure S3). These chromosomal patterns support previous evidence that parts of three linkage groups in *S. vulgaris* were translocated to the pseudo-autosomal region in *S. latifolia* through a double translocation event (Bergero et al., 2013). Our results support previous studies showing expansion of the PAR region in *S. latifolia* (Bergero et al., 2013; Campos et al., 2017) and newly in *S. dioica* (Figure 3). Alternatively, rearrangements could be accompanied by whole chromosome fusion(s) as documented in other species. In Japan Sea stickleback fish, an ancestral Y chromosome fused with the autosome, forming a neo-Y chromosome and the X_1_X_2_Y sex determination system (compared to closely related Pacific Ocean stickleback with XY system) (Yoshida et al., 2014). In *Rumex hastatulus*, North Carolina male race possesses XY_1_Y_2_, having the older Y (ancestral) chromosome heterochromatinised, and the younger Y with translocated autosomal part (Grabowska-Joachimiak et al., 2015). In *Silene* species “fusion” scenario is unlikely since it usually influences basic karyotype chromosome number (all studied species have *n* = 12). Moreover, existing genetic maps do not suggest large scale fusion events during karyotype evolution in studied species (Bergero et al., 2013). The existence of telomere-like sequences in *S. latifolia* Y chromosome (Uchida et al., 2002) can be explained as remnant of chromosomal inversion as was demonstrated in some *Silene* species (Filatov, 2005; Zluvova et al., 2005). It was reported that such chromosomal rearrangements included at least one paracentric and one pericentric inversion on the Y (Hobza et al., 2007; Bergero and Charlesworth, 2009). On the other hand, additional rearrangements that would suggest alternative scenarios cannot be excluded. Based on deletion mapping one inversion also occurred during the formation of the Y (Kazama et al., 2016). Bergero et al. (2013) and Campos et al. (2017) showed that PAR expanded through two step translocations. Our data support such translocation by the existence of three pair of autosomes (in gynodioecious species) corresponding to sex chromosomes in *S. latifolia* and *S. dioica*. Since sex chromosomes in studied dioecious species originated from one of these three chromosomal pairs, we assume that two additional loci were translocated on proto sex chromosomes in *S. latifolia* and *S. dioica* during sex chromosome evolution (Figure 3).

We have also tested the robustness of oligo painting method to study the dynamics of sex chromosomes and PAR in early metaphase/prophase (Supplementary Figure S3). In prophase/early metaphase in which the chromosomal (spatial) resolution limit is 10 times higher than in the metaphase and chromosomes are 10 times more extended (reviewed in Figueroa and Bass, 2010), the strength of the signal is relatively low. Therefore, it will be necessary to use more cytogenetic markers (together with oligo sequences) for discrimination of relaxed chromosomes such as e.g. specific antibodies against synaptonemal complexes in meiosis as described in Hurel et al. (2018).

Our X chromosome-specific oligo probe serves as useful tool to study the evolution of sex chromosomes in *S. latifolia*, *S. dioica* and their relatives. Since the genome of *S. latifolia* is still not fully sequenced we would like to leave the possibility that some sequences targeted by the probe might occur more than once in the genome open (e.g. low repetitive or duplicated). Nevertheless, compared to previous attempts and labeling of the sex chromosomes in *Silene* species using e.g. unique BAC clones (Lengerova et al., 2004) or dissected chromosomal probes (Hobza et al., 2004), the oligo painting probe provides an unique signal on both sex chromosomes and is also suitable to study other related species. In addition, the probe simplifies future analysis of chromosome pairing and facilitates the study of the dynamics of the PAR region in interphase or during cell division.

## Data Availability Statement

All datasets generated for this study are included in the article/Supplementary Material.

## Author Contributions

VB, RČ, and RH conceived and designed the research. VB, RČ, EH, and DŠ conducted the experiments. DŠ and EH contributed the reagents. VB analyzed the data. VB wrote the manuscript. All the authors read and approved the manuscript.

## Conflict of Interest

The authors declare that the research was conducted in the absence of any commercial or financial relationships that could be construed as a potential conflict of interest.

## Footnotes

1 https://imagej.nih.gov/ij/plugins/

## Supplementary Material

FIGURE S1 The distribution of X chromosome-specific oligo probe in X chromosome genetic map in *S. latifolia.* The average coverage was chosen on 2–3 oligo sequences/kb (1.8–5.5). Total size of X chromosome is estimated around 400 Mb.

FIGURE S2 Distribution of X chromosome-specific oligo in interphase in *S. latifolia*, *S. dioica*, *S. vulgaris* and *S. maritima*. Oligo painting probe differentiates two sub-domains in *S. latifolia* **(a)** and *S. dioica* **(b)**, and three to six sub-domains in *S. vulgaris* **(c)** and *S. maritima* **(d)**. Each sub-domain is enlarged and marked by separated colors (white/yellow/orange) in merged channel. X43.1, a sub-telomeric probe, is presented only on sex chromosomes and autosomes in *S. latifolia* and *S. dioica*. Note the distribution of STAR-C in *S. vulgaris* and *S. maritima*. Scale bar = 10 μm.

FIGURE S3 Distribution of X chromosome-specific oligo probe on prophase and early metaphase chromosomes in *S. latifolia*, *S. dioica*, *S. vulgaris* and *S. maritima*. 12 988 has coverage 2.5–3 oligo sequences/kb, reaching 1.8–5.5 oligo sequences/kb on selected loci. Oligo painting probe (in red) hybridizes to very end of X and Y chromosomes in (sub)telomeres in *S. latifolia* and *S. dioica*. In *S. vulgaris* and *S. maritima*, the oligo painting probe labels six pairs of autosomes, hybridizing to their p-arm. Although the signal strength is weaker compared to condensed metaphase chromosomes, the oligo painting probe clearly marks sex chromosomes and autosomes in all studied species. Scale bar = 10 μm.

FIGURE S4 Distribution of the oligo painting probe in *S. latifolia* and *S. dioica* female karyotype. Oligo painting probe was hybridized on metaphase chromosomes and on interphase nuclei in *S. latifolia* **(a,c)** and in *S. dioica* **(b,d)**. The remnant of a cytoplasm (signal not attached to any chromosome) is visible in the bottom of the *S. dioica* **(b)**. Scale bar = 10 μm.

TABLE S1 Plant material.

TABLE S2 Oligo probe sequence scaffolds ID.

TABLE S3 The composition of the enzyme mix and hybridisation stringency.

## References

[B1] Bachtrog D. (2006). A dynamic view of sex chromosome evolution. *Curr. Opin. Genet. Dev.* 16 578–585. 10.1016/j.gde.2006.10.007 17055249

[B2] Bachtrog D. Jensen J. D. Zhang Z. (2009). Accelerated adaptive evolution on a newly formed X chromosome. *PLoS Biol.* 7:e1000082. 10.1371/journal.pbio.1000082 19402745 PMC2672600

[B3] Bačovský V. Hobza R. Vyskot B. (2018). Technical review: cytogenetic tools for studying mitotic. *Methods Mol Biol.* 1675 509–535. 10.1007/978-1-4939-7318-7-30 29052211

[B4] Bačovský V. Houben A. Kumke K. Hobza R. (2019). The distribution of epigenetic histone marks differs between the X and Y chromosomes in *Silene latifolia*. *Planta* 250 487–494. 10.1007/s00425-019-03182-7 31069521

[B5] Bergero R. Charlesworth D. (2009). The evolution of restricted recombination in sex chromosomes. *Trends Ecol. Evol.* 24 94–102. 10.1016/j.tree.2008.09.010 19100654

[B6] Bergero R. Charlesworth D. (2011). Preservation of the Y Transcriptome in a 10-Million-year-old plant sex chromosome system. *Curr. Biol.* 21 1470–1474. 10.1016/j.cub.2011.07.032 21889891

[B7] Bergero R. Charlesworth D. Filatov D. A. Moore R. C. (2008a). Defining regions and rearrangements of the *Silene latifolia* Y chromosome. *Genetics* 178 2045–2053. 10.1534/genetics.107.084566 18245827 PMC2323795

[B8] Bergero R. Forrest A. Charlesworth D. (2008b). Active miniature transposons from a plant genome and its nonrecombining Y chromosome. *Genetics* 178 1085–1092. 10.1534/genetics.107.081745 18245352 PMC2248354

[B9] Bergero R. Qiu S. Forrest A. Borthwick H. Charlesworth D. (2013). Expansion of the pseudo-autosomal region and ongoing recombination suppression in the *Silene latifolia* sex chromosomes. *Genetics* 194 673–686. 10.1534/genetics.113.150755 23733786 PMC3697972

[B10] Bernasconi G. Antonovics J. Biere A. Charlesworth D. Delph L. F. Filatov D. (2009). Silene as a model system in ecology and evolution. *Heredity* 103 5–14. 10.1038/hdy.2009.34 19367316

[B11] Braz G. T. He L. Zhao H. Zhang T. Semrau K. Rouillard J.-M. (2018). Comparative oligo-FISH mapping: an efficient and powerful methodology to reveal karyotypic and chromosomal evolution. *Genetics* 208 513–523. 10.1534/genetics.117.300344 29242292 PMC5788518

[B12] Campos J. L. Qiu S. Guirao-Rico S. Bergero R. Charlesworth D. (2017). Recombination changes at the boundaries of fully and partially sex-linked regions between closely related Silene species pairs. *Heredity* 118 395–403. 10.1038/hdy.2016.113 27827389 PMC5345606

[B13] Cermak T. Kubat Z. Hobza R. Koblizkova A. Widmer A. Macas J. (2008). Survey of repetitive sequences in *Silene latifolia* with respect to their distribution on sex chromosomes. *Chromosom. Res.* 16 961–976. 10.1007/s10577-008-1254-2 18853265

[B14] Charlesworth D. (2016). Plant sex chromosomes. *Annu. Rev. Plant Biol.* 67 397–420. 10.1146/annurev-arplant-043015-111911 26653795

[B15] Charlesworth D. Charlesworth B. (2005). Sex chromosomes: evolution of the weird and wonderful. *Curr. Biol.* 15 R129–R131. 10.1016/j.cub.2005.02.011 15723783

[B16] Charlesworth D. Laporte V. (1998). The male-sterility polymorphism of Silene vulgaris: analysis of genetic dat from two populations and comparison with Thymus vulgaris. *Genetics* 150 1267–1282. 9799278 10.1093/genetics/150.3.1267 PMC1460393

[B17] Cui C. Shu W. Li P. (2016). Fluorescence in situ hybridization: cell-based genetic diagnostic and research applications. *Front. Cell Dev. Biol.* 4:89. 10.3389/fcell.2016.00089 27656642 PMC5011256

[B18] Darolti I. Wright A. E. Sandkam B. A. Morris J. Bloch N. I. Farré M. (2019). Extreme heterogeneity in sex chromosome differentiation and dosage compensation in livebearers. *Proc. Natl. Acad. Sci. U.S.A.* 116 19031–19036. 10.1073/pnas.1905298116 31484763 PMC6754558

[B19] Figueroa D. M. Bass H. W. (2010). A historical andmodern perspective on plant cytogenetics. *Briefings Funct. Genom. Proteom.* 9 95–102. 10.1093/bfgp/elp058 20110271

[B20] Filatov D. A. (2005). Evolutionary history of *Silene latifolia* sex chromosomes revealed by genetic mapping of four genes. *Genetics* 170 975–979. 10.1534/genetics.104.037069 15834147 PMC1450409

[B21] Filatov D. A. Howell E. C. Groutides C. Armstrong S. J. (2009). Recent spread of a retrotransposon in the *Silene latifolia* genome, apart from the Y chromosome. *Genetics* 181 811–817. 10.1534/genetics.108.099267 19064703 PMC2644968

[B22] Grabowska-Joachimiak A. Kula A. Książczyk T. Chojnicka J. Sliwinska E. Joachimiak A. J. (2015). Chromosome landmarks and autosome-sex chromosome translocations in *Rumex hastatulus*, a plant with XX/XY1Y2 sex chromosome system. *Chromosom. Res.* 23 187–197. 10.1007/s10577-014-9446-4 25394583 PMC4430600

[B23] Han Y. Zhang T. Thammapichai P. Weng Y. Jiang J. (2015). Chromosome-specific painting in *Cucumis* species using bulked oligonucleotides. *Genetics* 200 771–779. 10.1534/genetics.115.177642 25971668 PMC4512542

[B24] He L. Braz G. T. Torres G. A. Jiang J. (2018). Chromosome painting in meiosis reveals pairing of specific chromosomes in polyploid *Solanum* species. *Chromosoma* 127 505–513. 10.1007/s00412-018-0682-9 30242479

[B25] Hobza R. Hudzieczek V. Kubat Z. Cegan R. Vyskot B. Kejnovsky E. (2018). Sex and the flower – developmental aspects of sex chromosome evolution. *Ann. Bot.* 122 1085–1101. 10.1093/aob/mcy130 30032185 PMC6324748

[B26] Hobza R. Kejnovsky E. Vyskot B. Widmer A. (2007). The role of chromosomal rearrangements in the evolution of *Silene latifolia* sex chromosomes. *Mol. Genet. Genomics* 278 633–638. 10.1007/s00438-007-0279-0 17671795

[B27] Hobza R. Lengerova M. Cernohorska H. Rubes J. Vyskot B. (2004). FAST-FISH with laser beam microdissected DOP-PCR probe distinguishes the sex chromosomes of *Silene latifolia*. *Chromosom. Res.* 12 245–250. 10.1023/B:CHRO.0000021929.97208.1c 15125638

[B28] Hobza R. Vyskot B. (2007). Laser microdissection-based analysis of plant sex chromosomes. *Methods Cell Biol.* 82 433–453. 10.1016/S0091-679X(06)82015-7 17586267

[B29] Huber D. Voith von Voithenberg L. Kaigala G. V. (2018). Fluorescence in situ hybridization (FISH): history, limitations and what to expect from micro-scale FISH? *Micro Nano Eng.* 1 15–24. 10.1016/j.mne.2018.10.006

[B30] Hughes J. F. Skaletsky H. Pyntikova T. Graves T. A. van Daalen S. K. M. Minx P. J. (2010). Chimpanzee and human Y chromosomes are remarkably divergent in structure and gene content. *Nature* 463 536–539. 10.1038/nature08700 20072128 PMC3653425

[B31] Hurel A. Phillips D. Vrielynck N. Mézard C. Grelon M. Christophorou N. (2018). A cytological approach to studying meiotic recombination and chromosome dynamics in Arabidopsis thaliana male meiocytes in three dimensions. *Plant J.* 95 385–396. 10.1111/tpj.13942 29681056

[B32] Jiang J. (2019). Fluorescence in situ hybridization in plants: recent developments and future applications. *Chromosom. Res.* 27 153–165. 10.1007/s10577-019-09607-z 30852707

[B33] Kazama Y. Ishii K. Aonuma W. Ikeda T. Kawamoto H. Koizumi A. (2016). A new physical mapping approach refines the sex-determining gene positions on the *Silene latifolia* Y-chromosome. *Sci. Rep.* 6:18917. 10.1038/srep18917 26742857 PMC4705512

[B34] Kejnovsky E. Hobza R. Cermak T. Kubat Z. Vyskot B. (2009). The role of repetitive DNA in structure and evolution of sex chromosomes in plants. *Heredity* 102 533–541. 10.1038/hdy.2009.17 19277056

[B35] Kejnovský E. Michalovova M. Steflova P. Kejnovska I. Manzano S. Hobza R. (2013). Expansion of microsatellites on evolutionary young Y chromosome. *PLoS One* 8:45519. 10.1371/journal.pone.0045519 23341866 PMC3547029

[B36] Krasovec M. Chester M. Ridout K. Filatov D. A. (2018). The mutation rate and the age of the sex chromosomes in *Silene latifolia*. *Curr. Biol.* 28 1832.e–1838.e. 10.1016/j.cub.2018.04.069 29804812

[B37] Kubat Z. Zluvova J. Vogel I. Kovacova V. Cermak T. Cegan R. (2014). Possible mechanisms responsible for absence of a retrotransposon family on a plant Y chromosome. *New Phytol.* 202 662–678. 10.1111/nph.12669 24456522

[B38] Lengerova M. Kejnovsky E. Hobza R. Macas J. Grant S. R. Vyskot B. (2004). Multicolor FISH mapping of the dioecious model plant, *Silene latifolia*. *Theor. Appl. Genet.* 108 1193–1199. 10.1007/s00122-003-1568-6 14727034

[B39] Mathur J. Griffiths S. Barton K. Schattat M. H. (2012). Chapter eight - green-to-red photoconvertible meosfp-aided live imaging in plants. *Methods Enzymol.* 504 163–181. 10.1016/B978-0-12-391857-4.00008-2 22264534

[B40] Ming R. Wang J. Moore P. H. Paterson A. H. (2007). Sex chromosomes in flowering plants. *Am. J. Bot.* 94 141–150. 10.3732/ajb.94.2.141 21642216

[B41] Muyle A. Shearn R. Marais G. A. B. (2017). The evolution of sex chromosomes and dosage compensation in plants. *Genome Biol. Evol.* 9 627–645. 10.1093/gbe/evw282 28391324 PMC5629387

[B42] Pan W. H. Houben A. Schlegel R. (1993). Highly effective cell synchronization in plant roots by hydroxyurea and amiprophos-methyl or colchicine. *Genome* 36 387–390. 10.1139/g93-053 18469996

[B43] Papadopulos A. S. T. Chester M. Ridout K. Filatov D. A. (2015). Rapid Y degeneration and dosage compensation in plant sex chromosomes. *Proc. Natl. Acad. Sci. U.S.A.* 112 13021–13026. 10.1073/pnas.1508454112 26438872 PMC4620866

[B44] Rodríguez Lorenzo J. L. Hobza R. Vyskot B. (2018). DNA methylation and genetic degeneration of the Y chromosome in the dioecious plant *Silene latifolia*. *BMC Genomics* 19:540. 10.1186/s12864-018-4936-y 30012097 PMC6048894

[B45] Runyeon H. Prentice H. C. (1997). Genetic differentiation in the Bladder campions, *Silene vulgaris* and *S. uniflora* (Caryophyllaceae), in Sweden. *Biol. J. Linn. Soc.* 61 559–584. 10.1111/j.1095-8312.1997.tb01807.x

[B46] Schubert V. Ruban A. Houben A. (2016). Chromatin ring formation at plant centromeres. *Front. Plant Sci.* 7:28. 10.3389/fpls.2016.00028 26913037 PMC4753331

[B47] Šimoníková D. Němečková A. Karafiátová M. Uwimana B. Swennen R. Doležel J. (2019). Chromosome painting facilitates anchoring reference genome sequence to chromosomes in situ and integrated karyotyping in banana (Musa Spp.). *Front. Plant Sci.* 10:1503. 10.3389/fpls.2019.01503 31824534 PMC6879668

[B48] Široký J. Lysák M. A. Doležel J. Kejnovský E. Vyskot B. (2001). Heterogeneity of rDNA distribution and genome size in *Silene* spp. *Chromosom. Res.* 9 387–393. 10.1023/A:1016783501674 11448040

[B49] Stone J. D. Koloušková P. Sloan D. B. Štorchová H. (2017). Non-coding RNA may be associated with cytoplasmic male sterility in *Silene vulgaris*. *J. Exp. Bot.* 68 1599–1612. 10.1093/jxb/erx057 28369520 PMC5444436

[B50] Tang S. Tang Z. Qiu L. Yang Z. Li G. Lang T. (2018). Developing new oligo probes to distinguish specific chromosomal segments and the A, B, D genomes of wheat (*Triticum aestivum* L.) Using ND-FISH. *Front. Plant Sci.* 9:1104. 10.3389/fpls.2018.01104 30093909 PMC6070686

[B51] Uchida W. Matsunaga S. Sugiyama R. Shibata F. Kazama Y. Miyazawa Y. (2002). Distribution of interstitial telomere-like repeats and their adjacent sequences in a dioecious plant, *Silene latifolia*. *Chromosoma* 111 313–320. 10.1007/s00412-002-0213-5 12474060

[B52] Vyskot B. Hobza R. (2015). The genomics of plant sex chromosomes. *Plant Sci.* 236 126–135. 10.1016/j.plantsci.2015.03.019 26025526

[B53] Xin H. Zhang T. Han Y. Wu Y. Shi J. Xi M. (2018). Chromosome painting and comparative physical mapping of the sex chromosomes in *Populus tomentosa* and *Populus deltoides*. *Chromosoma* 127 313–321. 10.1007/s00412-018-0664-y 29520650

[B54] Yoshida K. Makino T. Yamaguchi K. Shigenobu S. Hasebe M. Kawata M. (2014). Sex chromosome turnover contributes to genomic divergence between incipient stickleback species. *PLoS Genet.* 10:e1004223. 10.1371/journal.pgen.1004223 24625862 PMC3953013

[B55] Zluvova J. Georgiev S. Janousek B. Charlesworth D. Vyskot B. Negrutiu I. (2007). Early events in the evolution of the *Silene latifolia* Y chromosome: male specialization and recombination arrest. *Genetics* 177 375–386. 10.1534/genetics.107.071175 17603119 PMC2013713

[B56] Zluvova J. Janousek B. Negrutiu I. Vyskot B. (2005). Comparison of the X and Y chromosome organization in *Silene latifolia*. *Genetics* 170 1431–1434. 10.1534/genetics.105.040444 15879508 PMC1451171

