# Supplementary figures and images for: The Formation of Sex Chromosomes in Silene latifolia and S. dioica Was Accompanied by Multiple Chromosomal Rearrangements
Source: Front Plant Sci. 2020 Feb 28;11:205. doi: 10.3389/fpls.2020.00205 (PMC7059608; doi:10.3389/fpls.2020.00205)

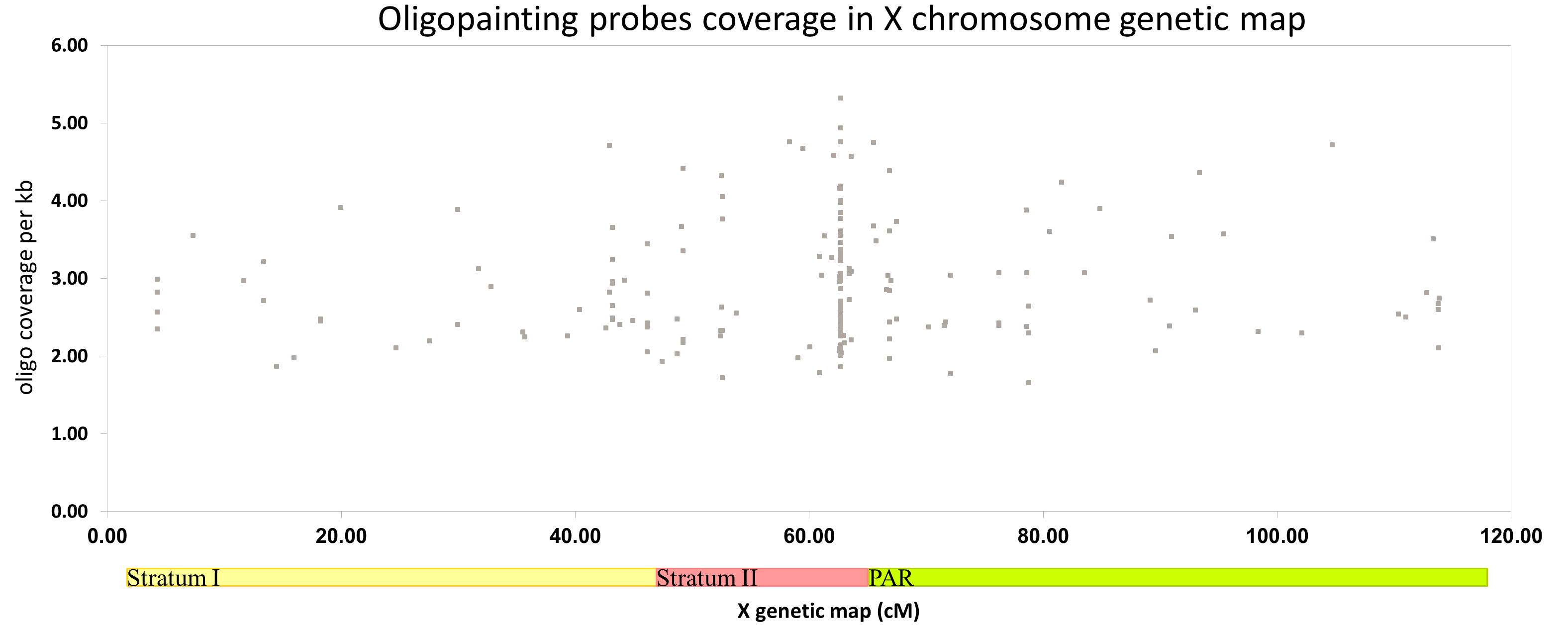

Supplement: FIGURE S1 — The distribution of X chromosome-specific oligo probe in X chromosome genetic map in S. latifolia. The average coverage was chosen on 2–3 oligo sequences/kb (1.8–5.5). Total size of X chromosome is estimated around 400 Mb. [file Image_1.TIF]

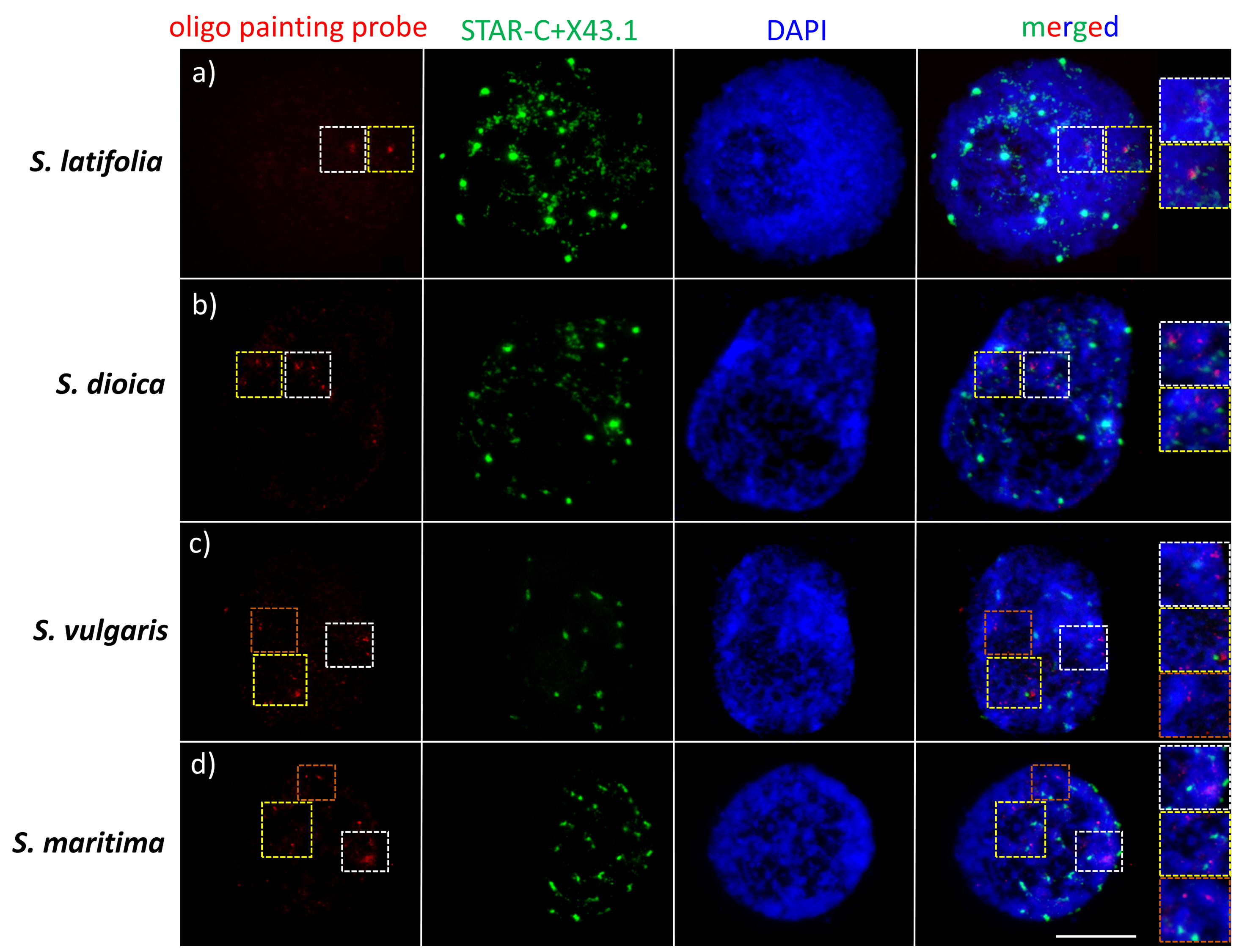

Supplement: FIGURE S2 — Distribution of X chromosome-specific oligo in interphase in S. latifolia, S. dioica, S. vulgaris and S. maritima. Oligo painting probe differentiates two sub-domains in S. latifolia (a) and S. dioica (b), and three to six sub-domains in S. vulgaris (c) and S. maritima (d). Each sub-domain is enlarged and marked by separated colors (white/yellow/orange) in merged channel. X43.1, a sub-telomeric probe, is presented only on sex chromosomes and autosomes in S. latifolia and S. dioica. Note the distribution of STAR-C in S. vulgaris and S. maritima. Scale bar = 10 μm. [file Image_2.tif]

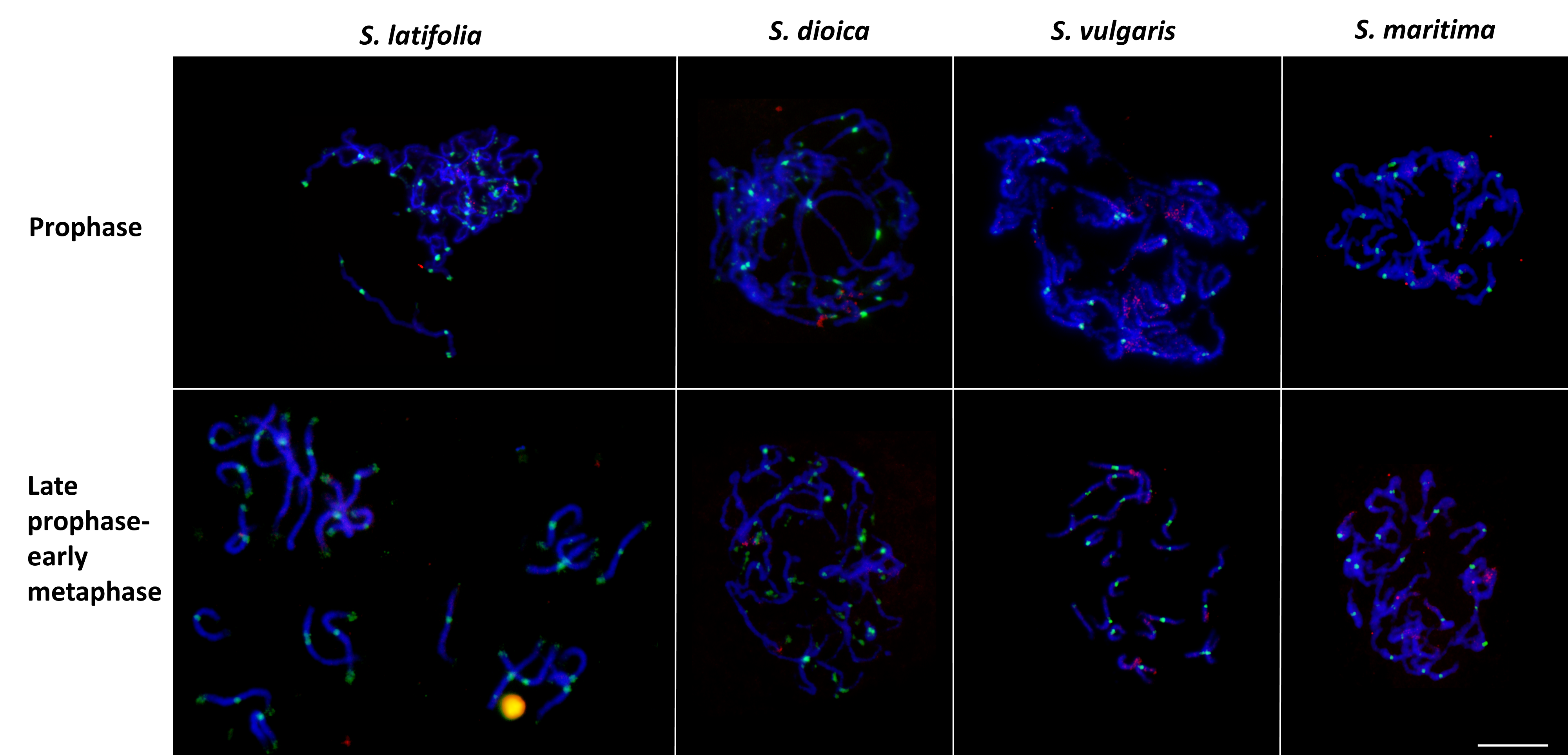

Supplement: FIGURE S3 — Distribution of X chromosome-specific oligo probe on prophase and early metaphase chromosomes in S. latifolia, S. dioica, S. vulgaris and S. maritima. 12 988 has coverage 2.5–3 oligo sequences/kb, reaching 1.8–5.5 oligo sequences/kb on selected loci. Oligo painting probe (in red) hybridizes to very end of X and Y chromosomes in (sub)telomeres in S. latifolia and S. dioica. In S. vulgaris and S. maritima, the oligo painting probe labels six pairs of autosomes, hybridizing to their p-arm. Although the signal strength is weaker compared to condensed metaphase chromosomes, the oligo painting probe clearly marks sex chromosomes and autosomes in all studied species. Scale bar = 10 μm. [file Image_3.TIF]

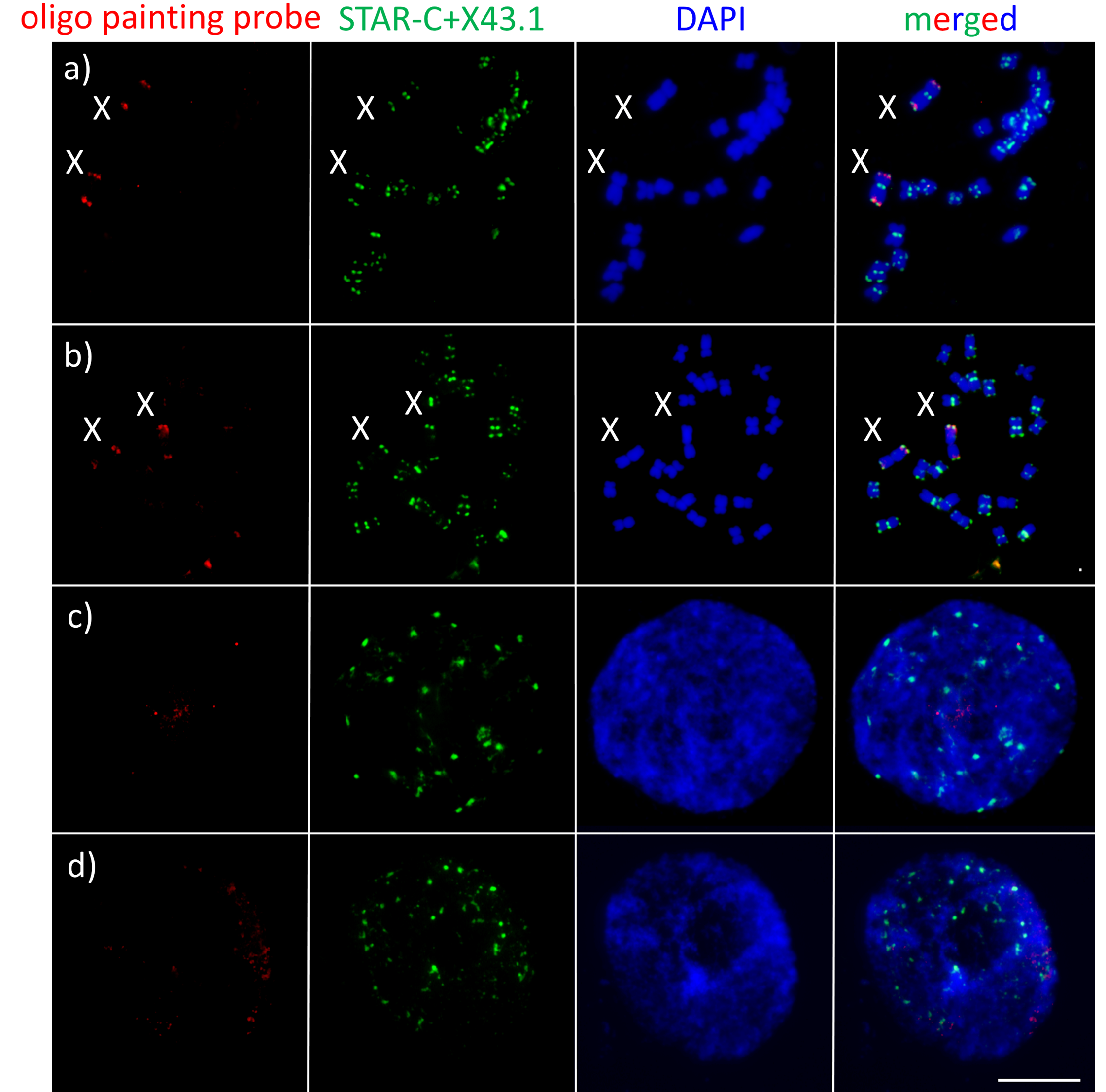

Supplement: FIGURE S4 — Distribution of the oligo painting probe in S. latifolia and S. dioica female karyotype. Oligo painting probe was hybridized on metaphase chromosomes and on interphase nuclei in S. latifolia (a,c) and in S. dioica (b,d). The remnant of a cytoplasm (signal not attached to any chromosome) is visible in the bottom of the S. dioica (b). Scale bar = 10 μm. [file Image_4.tif]
