# Supplementary material for: The Formation of Sex Chromosomes in Silene latifolia and S. dioica Was Accompanied by Multiple Chromosomal Rearrangements
Source: Front Plant Sci. 2020 Feb 28;11:205. doi: 10.3389/fpls.2020.00205 (PMC7059608; doi:10.3389/fpls.2020.00205)
Supplement: TABLE S1 — Plant material. [file Table_1.DOCX]

Table S1 – Plant material

| Species | Population | No. of chromosomes | Sex determination | GPS location |
| --- | --- | --- | --- | --- |
| *S. latifolia* | U16* | 24 | XY in males, XX in females | (Bačovský et al. 2019) |
| *S. dioica* | Tišnov (TIS) | 24 | XY in males, XX in females | 49°22'00.2"N 16°25'25.2"E |
| *S. vulgaris* | Čertovica (CERT) | 24 | Herm** | 48°54'27.5"N 19°44'03.4"E |
| *S. maritima* | Bratislava (BRA) | 24 | Herm** | collection of seeds of Institute of Biophysics of the Czech Academy of Sciences |

*population made by 16 generations of full-sib mating

**gynodioecious species
